# Maternal Obesity Increases Oxidative Stress in Placenta and It Is Associated With Intestinal Microbiota

**DOI:** 10.3389/fcimb.2021.671347

**Published:** 2021-08-23

**Authors:** Chengjun Hu, Yingli Yan, Fengjie Ji, Hanlin Zhou

**Affiliations:** Tropical Crops Genetic Resources Institute, Chinese Academy of Tropical Agricultural Sciences, Haikou, China

**Keywords:** intestinal microbiota, mitochondrial biogenesis, obesity, oxidative stress, placenta

## Abstract

Maternal obesity induces placental dysfunction and intestinal microbial dysbiosis. However, the associations between intestinal microbiota and placental dysfunction are still unclear. In the present study, a gilt model was used to investigate the role of maternal obesity on placental oxidative stress, mitochondrial function, and fecal microbiota composition, meanwhile identifying microbiota markers associated with placental oxidative stress. Twenty gilts were divided into two groups based on their backfat thickness on parturition day: namely Con group (average backfat thickness = 33 mm), and Obese group (average backfat thickness = 39 mm). The results showed that Obese group was lower than Con group in the birth weight of piglets. Compared with the Con group, the Obesity group exhibited an increased oxidative damage and inflammatory response in placenta, as evidenced by the increased concentrations of placental reactive oxygen species (ROS), protein carboxyl, and interleukin-6 (IL-6). Obesity group was lower than Con group in the concentrations of placental adenosine triphosphate, citrate synthase, and complex I activity. In addition, lower propionate level and Bacteroidetes abundance in feces were seen in the Obese Group. Furthermore, the concentrations of placental ROS, protein carboxyl, and IL-6 were positively correlated with the abundance of *Christensenellaceae_*R-7_group and negatively correlated with that of *norank_f_Bacteroidales*_S24-7_group. In conclusion, these findings suggest that maternal obesity might impair oxidative and inflammatory response in placenta through modulating intestinal microbiota composition.

## Introduction

Over the past few decades, the incidence of obesity has increased dramatically worldwide (Ng et al., 2014), which is one of the major risk factors for diseases, such as type-2 diabetes, inflammation, and cardiovascular diseases (Zhang et al., 2018). Specially, the prevalence of obesity among women is higher than that among men, which is 40.4% compared with 35% (Tauqeer et al., 2018). The number of obese or overweight women even increases during pregnancy, which induces placental dysfunction (Myatt and Maloyan, 2016; Godfrey et al., 2017). However, its mechanisms are still unclear.

Gut microbiota plays a vital role in the placental development. For instance, through transferring the microbiota from patients with pre-eclampsia to mice, the ratio of labyrinth and junctional zones was increased and the placental weight was decreased (Chen et al., 2020). However, with the current evidence, the change of placental function contributed by bacterial species is still unclear. Short-chain fatty acids (SCFAs), products of intestinal microbiota, not only serve as an energy source for hosts (Zhi et al., 2019), but also play a vital role in placental development. For instance, butyrate induces trophoblast differentiation and syncytiotrophoblast formation in early gestation (Kumar et al., 2015); and propionate blocks the secretion of pro-inflammatory cytokines and chemokines in placenta (Roy et al., 2020). Given SCFAs function as a link between the microbiota and the host, understanding the dynamic changes of SCFAs in obesity will help us have a better understanding of the relationship between intestinal microbiota and placental function.

Our previous study shows that maternal obesity increases oxidative stress and impairs mitochondrial function in placenta (Hu et al., 2019a). However, the relationship among the intestinal microbiota, placental oxidative stress, inflammatory response, and mitochondrial function has not been clarified so far. Gilt is an animal commonly used in human biomedical studies (Song et al., 2018). Therefore, gilts with a high backfat thickness were used as an obesity model to capture the changed microbiota involved in placental oxidative stress in obesity.

## Materials and Methods

### Animals and Experimental Treatments

**Figure 1:**
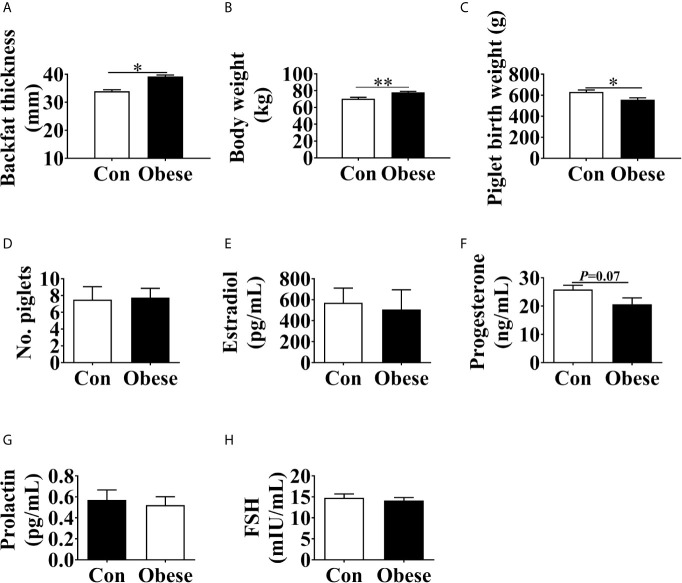
Phenotype changes in gilts with different backfat thickness. Maternal backfat thickness **(A)** and body weight **(B)** at parturition day. **(C)** Piglet birth weight. **(D)** Number of piglets at birth. The concentrations of estradiol **(E)**, progesterone **(F)**, prolactin **(G)**, prolactin, and FSH **(H)** in maternal serum. Values are mean ± SEM, n = 10. “*” and “**” indicate 0.01 < P ≤ 0.05 and 0.001 < P ≤ 0.01, respectively.

After mating, gilts were housed individually in a stall (1.8 × 0.7 × 1.0 m) and fed twice at 7:00 and 17:00 daily. The animals had ad libitum access to water through nipple drinkers. On parturition day, the body weight of gilts was weighed and their backfat thickness was measured at position P2 using ultrasonography (Renco Lean-Meater^®^, Minneapolis, MN, USA). Then the gilts were moved to the farrowing rooms and housed individually in slatted farrowing crates (2.0 m × 1.5 m). The total number of piglets born was recorded and their weight was measured within 6 h after birth. The average backfat thickness of the gilts was 36.61 ± 0.83 mm (means± standard error). Therefore, the gilts were assigned to two groups according to their backfat thickness: average backfat thickness = 33 mm (Con group) and average backfat thickness = 39 mm (Obese group) (**Figure 1A**).

### Sample Collection

Eight gilts were randomly selected from each group for sample collection. On parturition day, blood samples were collected from the ear vein through 10 mL centrifuge tubes and were centrifuged at 3,000 × g and 4°C for 15 min to obtain the serum. After the placentae were expelled, those of piglets with an average birth weight were obtained and separated from endometrium, after which about 3 g of placental samples (3 to 5 cm from the cord insertion point) were collected and quickly snap-frozen in liquid nitrogen. Colonic fecal samples were collected through massaging the rectum of those gilts on parturition day (Wang et al., 2019), which was then stored at -80°C for further analyses.

### Determination of Serum Hormone Level

The concentrations of progesterone, follicle-stimulating hormone (FSH), estradiol (CSB-E06844p,Cusabio, Wuhan, China, https://www.cusabio.com/), and prolactin (SEKP-0022, Solarbio, Beijing, China) in serum were determined through Elisa kits according to the manufacturer’s instructions.

### Oxidative Stress Parameters

The levels of reactive oxygen species (ROS), malondialdehyde (MDA), superoxide dismutase (SOD), glutathione (GSH), and protein carbonyl in placentae were analyzed through commercial kits (E004-1-1,A003-1-2, A001-3-2,A006-2-1, A087-1-1,Nanjing Jiancheng Bioengineering Institute, Nanjing, China) respectively.

### Adenosine Triphosphate (ATP), Citrate Synthase, Complex I and III Activities

ATP level (S0026, Beyotime, Beijing, China), citrate synthase activity (A108-1-2, Nanjing Jiancheng Bioengineering Institute, Nanjing, China) as well as complex I and III activities (FHTA-2-Y, FHTC-2-Y, Cominbio Co., Suzhou, China) in placentae were determined through commercial kits. The above tests were performed according to the manufacturer’s instructions.

### The Levels of Interleukin-6 (IL-6) and Tumor Necrosis Factor (TNF-α) in Placentae

The concentrations of IL-6 and TNF-α in placentae were determined using Elisa kits (CSB-E06786p, CSB-E16980p, Cusabio, Wuhan, China) according to the manufacturer’s instructions.

### Short-Chain Fatty Acids (SCFAs) Concentration in Feces

The samples were analyzed through gas chromatography (Hu et al., 2019c). The feces were dissolved in dd H_2_O and mixed through a vortex. The solution mixture was centrifuged at 10000 rpm/min to obtain the supernatant fluid, which was mixed with 25% metaphosphoric acid solution (1 mL:0.25 mL) and was then centrifuged at 20,000 ×*g* and 4°C for 10 min. The supernatant was filtered through a 0.45-μm polysulfone membrane before further analyses.

### Real-Time Quantitative PCR

The mRNA levels of targets genes were analyzed as we described previously (Hu et al., 2019a). Primers selected for PCR analyses are listed in **Supplemental Table 1**.

### Western Blotting

Total protein in placentae was extracted through homogenizing and lysing in RIPA lysis buffer (P0013B, Beyotime, Shanghai, China) and its concentration was determined using BCA kits (P0009, Beyotime, Shanghai, China). Protein was separated with SDS-PAGE and blotting onto PVDF membranes. Blots were then incubated overnight at 4°C with the following primary antibodies: peroxisome proliferator-activated receptor γ coactivator-1 (PGC-1α) antibody (2178S, CST, USA) and β-Actin antibody (4970, CST, USA). The density of bands was quantified through Image J software (National Institutes of Health, Bethesda, MD).

### DNA Extraction and PCR Amplification

DNA was extracted from feces samples (n = 6/group) through the HiPure Stool DNA kit B (D3141-02, Magen, Shanghai, China) according to the manufacturer’s instructions. The V3-V4 region of the 16S rRNA gene was amplified through universal primers 515F and 806R. PCR was performed as was previously described (Hu et al., 2019b). The amplified PCR products were identified through agarose gel electrophoresis. Then these products were extracted, purified through the SanPrep DNA Gel Extraction kit (B518131-0050, Sangon Biotech, Shanghai, China), and quantified through the PicoGreen dsDNA assay kit (P7581, Invitrogen, Carlsbad, CA, USA). Purified products were subject to paired-end sequencing on the Illumina MiSeq platform (Illumina, Sand Diego, CA, USA) according to a commercial service provider (Shanghai Majorbio Bio-pharm Technology Co., Ltd, Shanghai, China).

### Bioinformatics Analysis

The raw Illumina fastq files were quality-filtered, de-multiplexed and analyzed through QIIME and FLASH (v1.2.11) software package to obtain an original spliced sequence. The raw tags were analyzed using Trimmomatic (v.0.30) and FLASH (v.1.2.11) software package to obtain high-quality clean reads. The criteria for low quality sequences filtration was described in our previous study (Hu et al., 2019b). The effective tags were clustered into operational taxonomic units (OTUs) with a similarity of 97% through USEARCH (v7.0.1090). Then the OTU sequences were taxonomically classified using the Ribosomal Database Project (RDP) Classifier based on the Greengene (V201305) reference database.

Alpha diversity of microbiota was estimated based on ACE, Chao 1, Simpson, and Shannon indices. The beta (β) diversity of microbiota among samples was analyzed through a principal coordinate analysis (PCoA) based on an unweighted UniFrac distance. Phylogenetic investigation of communities by reconstruction of unobserved states (PICRUSt) was employed to predict microbial functions.

### Statistical Analyses

Data was presented as mean ± SEM. Statistical analyses were performed using SPSS 20.0 (SPPS Inc., Chicago, IL) software. Differences between the two groups were analyzed using student’s t-test. The relative species abundance of gut microbial communities was analyzed through the Mann-Whitney U-test. Spearman’s correlation coefficient was used to investigate the associations among backfat thickness, SCFAs level, oxidative stress parameters, and the relative abundance of microbiota. Differences were considered statistically significant at *P* < 0.05.

## Results

### Piglet Birth Weight and Maternal Serum Hormone Level

Compared to Con group, Obese group exhibited an increase in body weight of gilts (**Figure 1B**) and a decrease in piglet birth weight (**Figure 1C**). No difference was observed in the number of piglets at birth among the two groups (**Figure 1D**). In addition, the Obese group had a decreasing trend (*P* = 0.07) in the maternal serum progesterone level (**Figure 1F**). However, the concentrations of estradiol, prolactin, and FSH in serum (**Figures 1E, G, H**) showed no difference among the two groups.

### Oxidative Stress Level in Placentae

**Figure 2:**
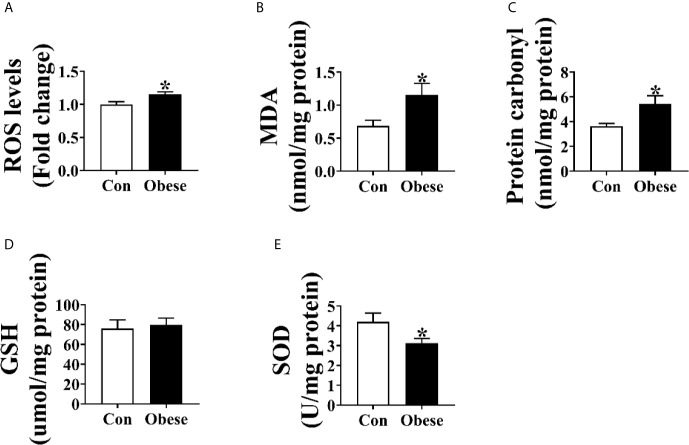
Oxidative stress levels in placentae. Placental ROS **(A)**, MDA **(B)**, protein carbonyl **(C)**, GSH **(D)**, and SOD **(E)** levels. Values are mean ± SEM, n = 8. “*” indicates statistically significant differences (*P* < 0.05).

As shown in **Figure 2**, placentae from Obese group were higher than that from Con group in the levels of ROS, MDA, and protein carbonyl (**Figures 2A–C**). Compared to Con group, there was an obvious decrease in the level of placental SOD in Obese group (**Figure 2E**). However, no difference was observed in the GSH level between Con and Obese groups (**Figure 2D**).

### Inflammatory Cytokine Level in Placentae

**Figure 3:**
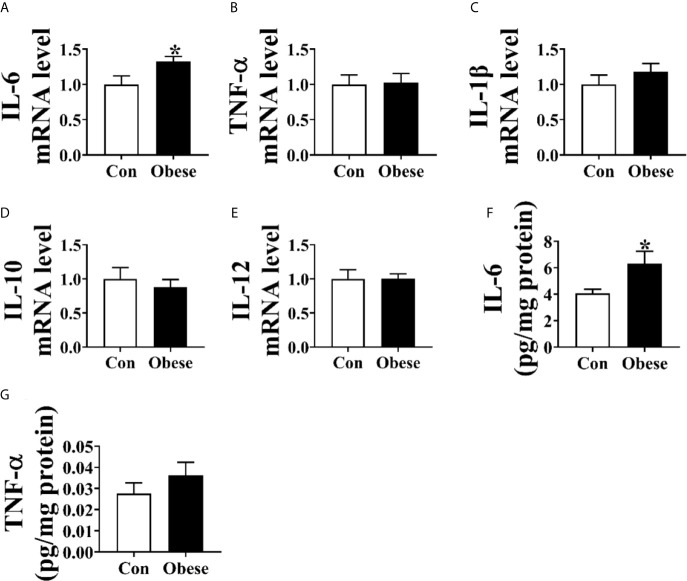
Placental inflammatory cytokine levels. **(A–E)** The mRNA levels of *IL-*6, *TNF-α*, *IL*-1β, *IL*-10, and *IL*-12 in placentae. The concentrations of IL-6 **(F)** and TNF-α **(G)** in placenta. Values are mean ± SEM, n = 8. “*” indicates statistically significant differences (*P* < 0.05).

As shown in **Figure 3**, Obese group was significantly lower than Con group in the level of placental IL-6 (**Figure 3A**) and mRNA of *IL-6* (**Figure 3C**). However, no difference was observed in the TNF-α level between Con and Obese groups (**Figure 3D**).

### Placental ATP Level and Mitochondrial Function

**Figure 4:**
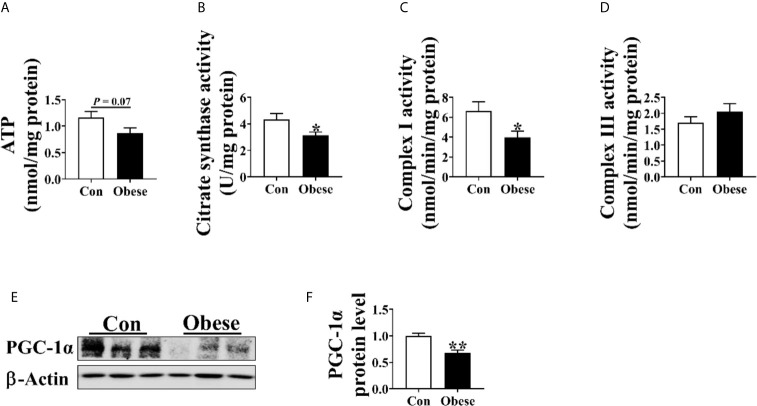
Mitochondrial function in placentae. Mitochondrial function was estimated by ATP **(A)** level, citrate synthase **(B)**, Complex I **(C)**, and III **(D)** activities. The protein level of PGC-1α in placentae **(E, F)**. Values are mean ± SEM, n = 8. “*” and “**” indicate P < 0.05 and < 0.01, respectively.

As shown in **Figure 4**, Obese group had a trend to decrease placental ATP level (**Figure 4A**) and was significantly lower than Con group in placental citrate synthase and complex I activity (**Figures 4B, C**). In addition, the protein level of PGC-1α in Obese group was lower than in Con group (**Figures 4E, F**).

### The Concentration of SCFAs in Feces

**Figure 5:**
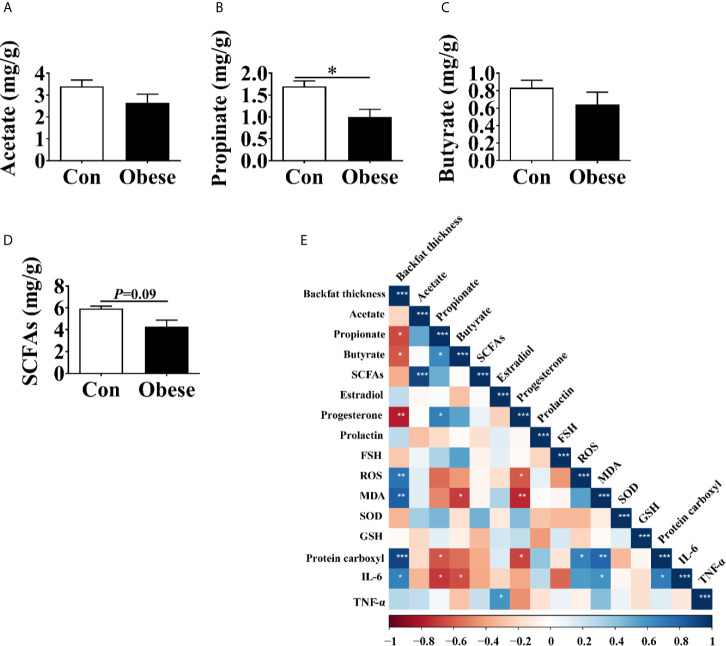
The concentrations of SCFAs in the feces of gilts. **(A–D)** Fecal acetate, propionate, and butyrate concentrations, respectively. **(E)** Correlation analysis of serum hormone, placental immune marker, antioxidant indexes, and fecal SCFAs levels. Data represent the means ± SEM. “*”, “**”, and “***” indicate P < 0.05, < 0.01, and < 0.001, respectively.

The concentration of SCFAs in feces was shown in **Figure 5**. When compared to Con group, the Obese group was seen to have a lower concentration of propionate in feces (**Figure 5B**). In addition, the Obese group displayed a lower trend (*P* = 0.09) in the concentration in the SCFAs (**Figure 5D**). The two groups showed no difference in the concentration of acetate or butyrate (**Figures 5A, C**).

The Spearman analysis showed that backfat thickness was negatively correlated with the concentrations of propionate, butyrate, progesterone, while was positively correlated with that of ROS, MDA, protein carboxyl, and IL-6 in placenta (**Figure 5E**), indicating that maternal obesity was positively correlated with placental oxidative stress.

The concentration of propionate was negatively correlated with that of protein carboxyl and IL-6 in placenta, while was positively correlated with that of progesterone. In addition, the concentration of serum progesterone was negatively correlated with that of ROS, MDA, and protein carboxyl in placenta.

### Diversity of Fecal Microbiota Communities

After size filtering, quality control, and chimera removal, an average of 39, 304 and 40, 224 sequences were obtained in Con and obese groups, respectively (**Supplemental Table 2**). The rarefaction curves indicated that almost all bacterial species were obtained (**Supplemental Figure 1**). 1,158 and 1,223 OUTs were obtained in Con and obese group, respectively, 1,055 of which were shared (**Supplemental Figure 2**).

**Figure 6:**
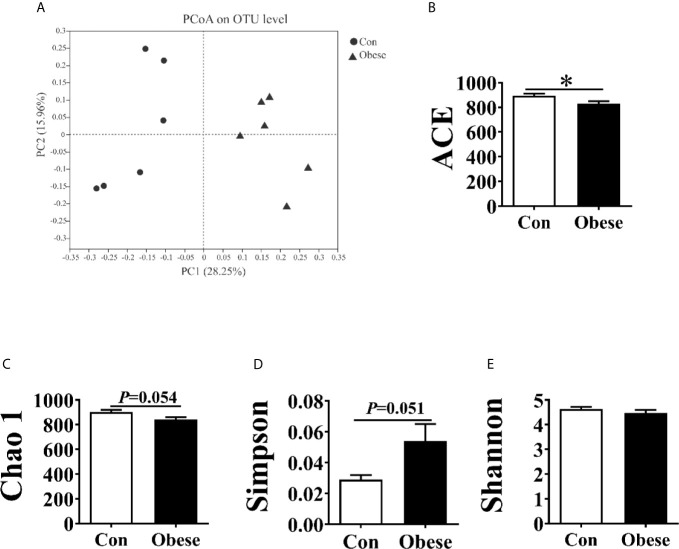
Alpha and beta diversities for the fecal microbiota community. **(A)** Principal coordinates analysis (PCoA, Bray–Curtis distance) plot of the fecal microbial community structure. **(B–E)**. The microbiota α diversity was estimated by ACE, Chao 1, Simpson, and Shannon index. “*” indicates statistically significant differences (P < 0.05).

The PCoA analysis showed that fecal samples tended to cluster according to groups (**Figure 6A**). α diversity of microbiota was estimated by ACE, Chao 1, Simpson and Shannon index. Compared to the Con group, the Obese group showed a lower ACE, Chao 1, and Simpson index (**Figures 6B–D**). However, the 2 groups showed no difference in the Shannon index (**Figure 6E**).

### Microbiota Community Composition

**Figure 7:**
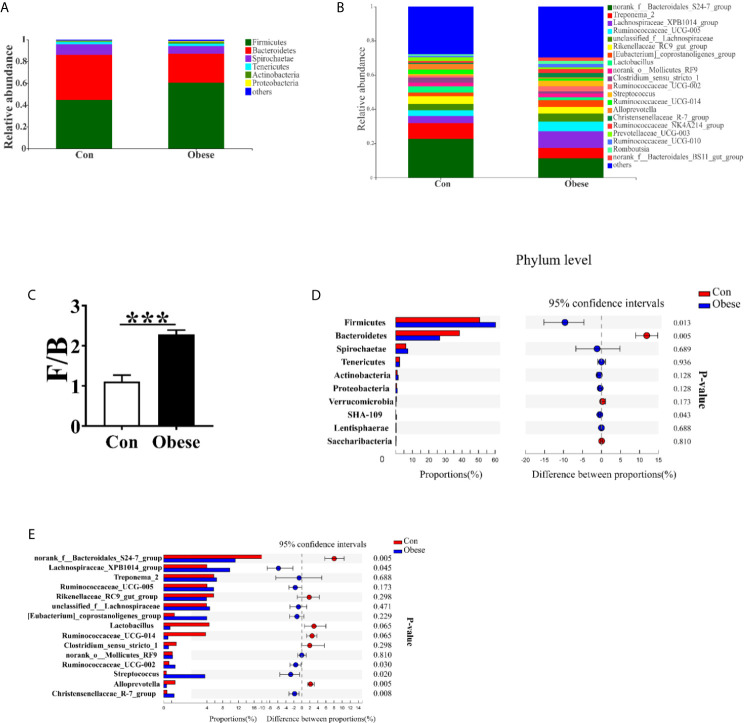
Microbial community composition. Distribution of microbiota composition at phylum **(A)** and genera levels **(B)**. **(C)** The ratio of Firmicutes to Bacteroidetes (F: B). Comparison of relative abundance at the phylum **(D)** and genus **(E)** levels between the two groups.

The most dominant phyla among microbiota communities (>1%) were Firmicutes, Bacteroidetes, Spirochaetes, Tenericutes, Actinobacteria, and Proteobacteria (**Figure 7A**); these microbiotas accounted for more than 96% of the total microbiota found in fecal samples. Obese group was higher than Con group in the Firmicutes: Bacteroidetes (F: B) ratio (**Figure 7C**). Compared to Con group, Obese group displayed a higher abundance of Firmicutes, and a lower abundance of Bacteroidetes (**Figure 7D**).

**Figure 7B** shows the abundance of the bacterial genera (>1%) in the two groups. The most dominant genus among microbiota communities were *norank_f_ Bacteroidetes_S*24-7*_*group, *Treponema*_2, *Lachnospiraceae_XPB*1014*_*group, *Ruminococcaceae_UCG*_005, and *unclassified_*f_ *Lachnospiraceae.* The abundances of *norank_f_ Bacteroidetes_S24-7_*group and *Alloprevotella* were lower, while that of *Lachnospiraceae_XPB1014_*group, *Ruminococcaceae_UCG*-002, *Streptococcus*, and *Christensenellaceae_R-7*_group were higher, in the Obese group than in the Con group (**Figure 7E**). In addition, the Obese group displayed a lower trend abundance of *lactobacillus* and *ruminococcaceae_UCG-*014.

### Correlation Between Microbial Community Composition, Oxidative Stress Index, Immune Marker, SCFAs, and Serum Hormone Level

**Figure 8:**
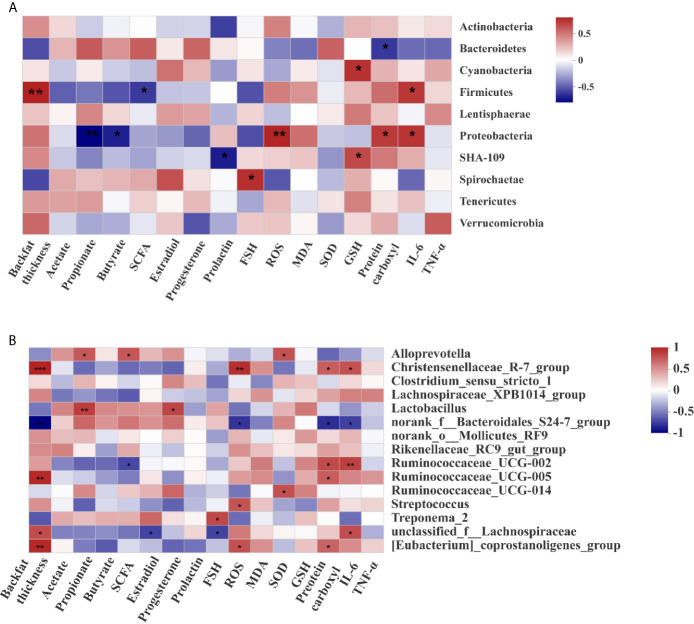
Correlations between the microbiota, backfat thickness, short-chain fatty acids, and hormone levels. Correlation analysis at the phylum **(A)** and genera **(B)** levels. “*”, “**”, and “***” indicate P < 0.05, < 0.01,and < 0.001 respectively.

At the phylum level (**Figure 8A**), the abundance of Firmicutes was positively correlated with backfat thickness and the concentration of IL-6 in placentae, while was negatively correlated with the concentration of SCFAs. The abundance of Bacteroidetes was negatively correlated with the concentration of protein carboxyl in placentae, and that of Proteobacteria was positively correlated with the concentrations of ROS, protein carboxyl, and IL-6 in placentae, while was negatively correlated with the concentrations of propionate and butyrate.

At the genus level (**Figure 8B**), the abundance of *Christensenellaceae_*R-7_group was positively correlated with backfat thickness, the concentrations of ROS, protein carboxyl, and IL-6. The abundance of *Ruminococcaceae_UCG*-005 was positively correlated with backfat thickness and the concentration of protein carboxyl, while that of *norank_f:Bacteroidales*_S24-7_group was negatively correlated with backfat thickness, the concentrations of placental ROS, protein carboxyl and IL-6. These results suggest that maternal obesity was positively correlated with the abundance of *Ruminococcaceae_UCG*-005, but negatively correlated with the abundance of *norank_f_Bacteroidales*_S24-7_group.

### Function Prediction of Microbiota Communities

PICRUSt was used to estimate the function of the fecal microbiota in gilts of the Con and Obese group. As shown in **Table 1**, microbiota from Obese gilts had a lower trend (*P* = 0.09) abundance of energy production and conversion, and displayed lower abundances of carbohydrate transport, metabolism, amino acid transport, and metabolism than that of gilts in the Con group.

**Table 1:** PICRUSt analysis of predictive metagenomics function of the fecal microbiota.

Description	CON	Obese	*P*-value
Translation, ribosomal structure, and biogenesis	4084414 ± 153262	3997426 ± 153313	0.70
RNA processing and modification	154 ± 22	389 ± 81	0.02
Transcription	2904158 ± 177021	3129631 ± 140965	0.34
Replication, recombination, and repair	2649832 ± 109236	2617864 ± 106529	0.84
Chromatin structure and dynamics	794 ± 111	998 ± 130	0.26
Cell cycle control, cell division, chromosome partitioning	602493 ± 26095	585050 ± 26259	0.65
Defense mechanisms	1096506 ± 55118	1120853 ± 44694	0.74
Signal transduction mechanisms	1643514 ± 64697	1664604 ± 63506	0.82
Cell wall/membrane/envelope biogenesis	3157429 ± 96701	2793209 ± 109657	0.03
Cell motility	437506 ± 31128	512648 ± 16134	0.06
Cytoskeleton	672 ± 146	967 ± 66	0.10
Extracellular structures	7 ± 3	5 ± 3	0.60
Intracellular trafficking, secretion, and vesicular transport	562194 ± 18826	554831 ± 20164	0.79
Posttranslational modification, protein turnover, chaperones	1524499 ± 49713	1432079 ± 49258	0.22
Energy production and conversion	2567778 ± 60845	2322549 ± 114626	0.09
Carbohydrate transport and metabolism	3128111 ± 70640	2698067 ± 79519	0.00
Amino acid transport and metabolism	4248646 ± 60952	3751842 ± 140894	0.01
Nucleotide transport and metabolism	1711731 ± 62403	1629905 ± 63121	0.38
Coenzyme transport and metabolism	1678606 ± 75618	1629827 ± 73324	0.65
Lipid transport and metabolism	1066536 ± 42025	1065261 ± 36048	0.98
Inorganic ion transport and metabolism	2351984 ± 102963	2298300 ± 87792	0.70
Secondary metabolites biosynthesis, transport, and catabolism	206926 ± 9555	190387 ± 8092	0.22
Function unknown	6627472 ± 327201	6695151 ± 247724	0.87

## Discussion

In the present study, a gilt obesity model was used to identify microbiota markers associated with placental oxidative stress. We found that gilts with a high backfat thickness showed a decrease in the birth weight of piglets. This result was in line with previous observation, where it was reported that maternal obesity led to a decrease in the birth weight of piglets (Zhou et al., 2018; Hu et al., 2019a). Hormone regulates fetal growth. Therefore, the levels of hormones in serum were measured. Here, no difference was observed in the concentrations of estradiol, prolactin, or FSH in the maternal serum of the two groups, while obese gilts showed a lower trend level in the serum progesterone, which was consistent with previous observation (Goh et al., 2016). Low serum progesterone was a risk factor for low birth weight (He et al., 2016), suggesting that a decreased birth weight induced by maternal obesity might attribute to the imbalance of progesterone secretion.

Placenta is the main tissue for progesterone secretion during pregnancy (López Bernal, 2007); in turn, progesterone supports placental growth and function (Mark et al., 2006). Evidences showed that maternal obesity impairs placental function (Hu et al., 2019a). In the present study, we showed that maternal obesity increased the oxidative stress levels in placenta, as evidenced by increased ROS level and the decreased SOD level, which was in line with a previous study showing that the placental ROS level was increased by maternal adiposity (Mele et al., 2014). Oxidative stress induces oxidative damage to lipid and protein through lipid and protein peroxidation (Dalle-Donne et al., 2003; Guéraud et al., 2010). A higher level of MDA and protein carbonyl in placenta further confirmed that the placenta of obese gilts was subjected to a higher oxidative stress. Mitochondria are the major ROS generation sites, and most of the ROS are generated by Complex I (Nishikawa et al., 2007; Kim and Byzova, 2014). In the present study, the activity of placental complex I was lower among obese gilts, suggesting that mitochondrial function might be functionally impaired, thus contributing to ROS generation. In addition, a decreased placental mitochondrial function was also observed among obese gilts, as evidenced by a decreased ATP level, citrate synthase activity, and the protein level of PGC-1α. In support of our view, an impaired mitochondrial function of human placentae with an increased maternal adiposity was also reported in a previous study (Mele et al., 2014). Overproduction of ROS can destroy normal placental functions (Wu et al., 2016). Therefore, we suspected that the reduction of progesterone induced by maternal obesity might be attributed to placenta dysfunction.

A previous study has showed that the intestinal microbiota played an important role in oxidative stress (Chen et al., 2020). Therefore, we firstly investigated the effects of maternal obesity on fecal microbiota composition. Our study showed that the microbial diversity was significantly decreased by obesity, as evidenced by ACE, Chao 1, and Simpson index. In addition, the Bacteroidetes and Firmicutes were the dominant phyla in feces. Firmicutes bacteria have a better capacity than Bacteroidetes bacteria in the ferment and metabolize carbohydrates as well as lipids, thus contributing to the development of obesity (Stojanov and Berlec, 2020). Gilts from Obese group displayed a higher abundance of Firmicutes and a lower abundance of Bacteroidetes, which was in line with a previous study showing that the relative proportion of Bacteroidetes was decreased among obese people in comparison with lean people (Ley et al., 2006). Consistence with the change of the abundances of Firmicutes and Bacteroidetes, the abundances of *norank_f_ Bacteroidetes_*S24-7_group and Alloprevotella (belonging phylum Bacteroidetes) were lower, and that of *Lachnospiraceae_XPB*1014_group, *Ruminococcaceae_UCG*-002, *Streptococcus*, and *Christensenellaceae_R*-7_group (belonging to Firmicutes) were higher in the Obese group than in the Con group. The relationship between microbiota composition and placental oxidative stress was revealed through Spearman analysis. We observed that the abundance of *Christensenellaceae_*R-7_group was positively correlated with the concentrations of ROS, protein carboxyl, and IL-6 in placentae, and that of *norank_f:Bacteroidales_*S24-7_group was negatively correlated with the levels of placental ROS, protein carboxyl, and IL-6. Changes in the intestinal microbiota was reported to induce oxidative stress. For instance, germ-free mice were infused intracolonically with fecal supernatants of acute ischemic stroke increased the levels of ROS and MDA (Xu et al., 2018); suppression of the gut microbiome ameliorates age-related arterial dysfunction and oxidative stress in mice (Brunt et al., 2019). Intestine microbiota also shapes host immune responses. Germ-free mice demonstrated that microbiota colonization in early life is necessary for development of the immune system, absence of microbiota resulted in a weakened capacity to fight off pathogenic bacteria (Sommer and Bäckhed, 2013). Therefore, we suspected that maternal obesity might induce placental oxidative damage and inflammatory response through modulating the abundance of *Christensenellaceae_*R-7_group and *norank_f:Bacteroidales_*S24-7_group. However, the mechanism needs further investigation.

SCFAs are a possible link between the intestinal microbiota and placenta (Kumar et al., 2015). Therefore, the concentration of SCFAs in feces was determined in the present study. Our data showed that the concentration of propionate was lower in Obese group than in Con group. In addition, the concentration of propionate was negatively correlated with that of protein carboxyl and IL-6 in placenta. Propionate treatment was shown to be effective against oxidative stress and ameliorate systemic inflammation (Hoyles et al., 2018; Bartolomaeus et al., 2019). Moreover, the secretion of pro-inflammatory cytokines and chemokines in placenta was blocked by a propionate treatment (Roy et al., 2020). Based on these lines of evidence available, we suspected that a decreased concentration of propionate might partially contribute to the development of placental oxidative stress. Interestingly, the concentration of propionate was positively correlated with that of serum progesterone. In line with our study, the infusion of propionate led to an increased concentration of plasma progesterone in a dairy heifer model (Bedford et al., 2018). These data indicated that a decreased propionate production might induce abnormal placental function, thus contributing to the decrease of progesterone secretion. Previous study showed that propionate and butyrate enhance mitochondrial biogenesis through extracellular signal-regulated kinases 1/2-dependent mechanism (Ribeiro et al., 2020), suggesting intestine microbiota modulate mitochondrial biogenesis thought regulating SCFAs production. Propionate is produced through the fermentation of dietary carbohydrate and amino acid by the intestinal microbiota (Neis et al., 2015). Therefore, we employed PICRUSt analysis to investigate the function of microbiota to determine the metabolic alterations caused by obesity. In the present study, microbiota in obesity gilts had a lower abundance in the transport and metabolism of carbohydrate and amino acid, indicating that a decreased carbohydrate and amino acid metabolism of microbiota might lead to the reduction of propionate production.

## Conclusions

We found that maternal obesity during late pregnancy increased placental oxidative damage, induced mitochondrial dysfunction, and decreased birth weight of newborn. We identified that the microbiota markers *Christensenellaceae_R-7*_group and *norank_f:Bacteroidales*_S24-7_group were associated with placental oxidative stress. The results of the present study contribute to the first evidence that the intestinal microbiota is an important mediator of obesity‐related placental oxidative stress and suggest that therapeutic strategies targeted at intestinal microbiota health may preserve placental function and reduce the risk of pregnancy syndromes in humans.

## Data Availability Statement

The datasets presented in this study can be found in online repositories. The names of the repository/repositories and accession number(s) can be found below: NCBI SRA [accession: SRR14292254–SRR14292265].

## Ethics Statement

This study was performed in accordance with the recommendations of the Guide for the Care and Use of Chinese Academy of Tropical Agricultural Sciences.

## Author Contributions

CH, YY and HZ contributed to the study design, conducted the animal experiments, and wrote the manuscript. CH and YY executed the lab analysis. YY and FJ performed the statistical analysis and HZ revised the paper. All authors contributed to the article and approved the submitted version.

## Funding

The present work was jointly supported by Central Public-interest Scientific Institution Basal Research Fund for Chinese Academy of Tropical Agricultural Sciences (No.1630032021004) and the Integrated Demonstration of Key Techniques for the Industrial Development of Featured Crops in Rocky Desertification Areasof Yunnan-Guangxi-Guizhou Provinces (SMH2019-2021).

## Conflict of Interest

The authors declare that the research was conducted in the absence of any commercial or financial relationships that could be construed as a potential conflict of interest.

## Publisher’s Note

All claims expressed in this article are solely those of the authors and do not necessarily represent those of their affiliated organizations, or those of the publisher, the editors and the reviewers. Any product that may be evaluated in this article, or claim that may be made by its manufacturer, is not guaranteed or endorsed by the publisher.

## References

[B1] Bartolomaeus H. Balogh A. Yakoub M. Homann S. Markó L. Höges S.. (2019). Short-Chain Fatty Acid Propionate Protects From Hypertensive Cardiovascular Damage. Circulation 139, 1407–1421. 10.1161/CIRCULATIONAHA.118.036652 30586752 PMC6416008

[B2] Bedford A. Beckett L. Hardin K. Dias N. W. Davis T. Mercadante V. R. G.. (2018). Propionate Affects Insulin Signaling and Progesterone Profiles in Dairy Heifers. Sci. Rep. 8, 17629. 10.1038/s41598-018-35977-1 30514961 PMC6279792

[B3] Brunt V. E. Gioscia-Ryan R. A. Richey J. J. Zigler M. C. Cuevas L. M. Gonzalez A.. (2019). Suppression of the Gut Microbiome Ameliorates Age-Related Arterial Dysfunction and Oxidative Stress in Mice. J. Physiol. 597, 2361–2378. 10.1113/JP277336 30714619 PMC6487935

[B4] Chen X. Li P. Liu M. Zheng H. He Y. Chen M. X.. (2020). Gut Dysbiosis Induces the Development of Pre-Eclampsia Through Bacterial Translocation. Gut 69, 513–522. 10.1136/gutjnl-2019-319101 31900289

[B5] Dalle-Donne I. Rossi R. Giustarini D. Milzani A. Colombo R. (2003). Protein Carbonyl Groups as Biomarkers of Oxidative Stress. Clin. Chim. Acta 329, 23–38. 10.1016/s0009-8981(03)00003-2 12589963

[B6] Godfrey K. M. Reynolds R. M. Prescott S. L. Nyirenda M. Jaddoe V. W. Eriksson J. G.. (2017). Influence of Maternal Obesity on the Long-Term Health of Offspring. Lancet Diabetes Endocrinol. 5, 53–64. 10.1016/S2213-8587(16)30107-3 27743978 PMC5245733

[B7] Goh J. Y. He S. Allen J. C. Malhotra R. Tan T. C. (2016). Maternal Obesity Is Associated With a Low Serum Progesterone Level in Early Pregnancy. Horm. Mol. Biol. Clin. Investig. 27, 97–100. 10.1515/hmbci-2015-0030 26751901

[B8] Guéraud F. Atalay M. Bresgen N. Cipak A. Eckl P. M. Huc L.. (2010). Chemistry and Biochemistry of Lipid Peroxidation Products. Free Radic. Res. 44, 1098–1124. 10.3109/10715762.2010.498477 20836659

[B9] He S. Allen J. C. Jr. Malhotra R. Østbye T. Tan T. C. (2016). Association of Maternal Serum Progesterone in Early Pregnancy With Low Birth Weight and Other Adverse Pregnancy Outcomes. J. Matern Fetal Neonatal Med. 29, 1999–2004. 10.3109/14767058.2015.1072159 26335272

[B10] Hoyles L. Snelling T. Umlai U. K. Nicholson J. K. Carding S. R. Glen R. C.. (2018). Microbiome-Host Systems Interactions: Protective Effects of Propionate Upon the Blood-Brain Barrier. Microbiome 6, 55. 10.1186/s40168-018-0439-y 29562936 PMC5863458

[B11] Hu C. Li F. Duan Y. Yin Y. Kong X. (2019b). Dietary Supplementation With Leucine or in Combination With Arginine Decreases Body Fat Weight and Alters Gut Microbiota Composition in Finishing Pigs. Front. Microbiol. 10, 1767. 10.3389/fmicb.2019.01767 31456756 PMC6700229

[B12] Hu C. Li F. Duan Y. Yin Y. Kong X. (2019c). Glutamic Acid Supplementation Reduces Body Fat Weight in Finishing Pigs When Provided Solely or in Combination With Arginine and It Is Associated With Colonic Propionate and Butyrate Concentrations. Food Funct. 10, 4693–4704. 10.1039/c9fo00520j 31298673

[B13] Hu C. Yang Y. Li J. Wang H. Cheng C. Yang L.. (2019a). Maternal Diet-Induced Obesity Compromises Oxidative Stress Status and Angiogenesis in the Porcine Placenta by Upregulating Nox2 Expression. Oxid. Med. Cell Longev 2019, 2481592. 10.1155/2019/2481592 31662816 PMC6791269

[B14] Kim Y. W. Byzova T. V. (2014). Oxidative Stress in Angiogenesis and Vascular Disease. Blood 123, 625–631. 10.1182/blood-2013-09-512749 24300855 PMC3907751

[B15] Kumar P. Thirkill T. L. Ji J. Monte L. H. Douglas G. C. (2015). Differential Effects of Sodium Butyrate and Lithium Chloride on Rhesus Monkey Trophoblast Differentiation. PloS One 10, e0135089. 10.1371/journal.pone.0135089 26266541 PMC4533975

[B16] Ley R. E. Turnbaugh P. J. Klein S. Gordon J. I. (2006). Microbial Ecology: Human Gut Microbes Associated With Obesity. Nature 444, 1022–1023. 10.1038/4441022a 17183309

[B17] López Bernal A. (2007). Overview. Preterm Labour: Mechanisms and Management. BMC Pregnancy Childbirth 7 (Suppl 1), S2. 10.1186/1471-2393-7-S1-S2 17570162 PMC1892059

[B18] Mark P. J. Smith J. T. Waddell B. J. (2006). Placental and Fetal Growth Retardation Following Partial Progesterone Withdrawal in Rat Pregnancy. Placenta 27, 208–214. 10.1016/j.placenta.2005.01.004 16338466

[B19] Mele J. Muralimanoharan S. Maloyan A. Myatt L. (2014). Impaired Mitochondrial Function in Human Placenta With Increased Maternal Adiposity. Am. J. Physiol. Endocrinol. Metab. 307, E419–E425. 10.1152/ajpendo.00025.2014 25028397 PMC4154072

[B20] Myatt L. Maloyan A. (2016). Obesity and Placental Function. Semin. Reprod. Med. 34, 42–49. 10.1055/s-0035-1570027 26734917

[B21] Neis E. P. Dejong C. H. Rensen S. S. (2015). The Role of Microbial Amino Acid Metabolism in Host Metabolism. Nutrients 7, 2930–2946. 10.3390/nu7042930 25894657 PMC4425181

[B22] Ng M. Fleming T. Robinson M. Thomson B. Graetz N. Margono C.. (2014). Global, Regional, and National Prevalence of Overweight and Obesity in Children and Adults During 1980-2013: A Systematic Analysis for the Global Burden of Disease Study 2013. Lancet 384, 766–781. 10.1016/S0140-6736(14)60460-8 24880830 PMC4624264

[B23] Nishikawa T. Kukidome D. Sonoda K. Fujisawa K. Matsuhisa T. Motoshima H.. (2007). Impact of Mitochondrial ROS Production on Diabetic Vascular Complications. Diabetes Res. Clin. Pract. Suppl 1, S41–S45. 10.1016/j.diabres.2007.01.031 17452060

[B24] Ribeiro M. F. Santos A. A. Afonso M. B. Rodrigues P. M. Sónia S. S. m Castro R. E.. (2020). Diet-Dependent Gut Microbiota Impacts on Adult Neurogenesis Through Mitochondrial Stress Modulation. Brain Commu 2, fcaa165. 10.1093/braincomms/fcaa165 PMC7780462 33426525

[B25] Roy R. Nguyen-Ngo C. Lappas M. (2020). Short-Chain Fatty Acids as Novel Therapeutics for Gestational Diabetes. J. Mol. Endocrinol. 65, 21–34. 10.1530/JME-20-0094 32580157

[B26] Sommer F. Bäckhed F. (2013). The Gut Microbiota - Masters of Host Development and Physiology. Nat. Rev. Microbiol. 11, 227–238. 10.1038/nrmicro2974 23435359

[B27] Song T. Lu J. Deng Z. Xu T. Yang Y. Wei H.. (2018). Maternal Obesity Aggravates the Abnormality of Porcine Placenta by Increasing N(6)-Methyladenosine. Int. J. Obes. 42, 1812–1820. 10.1038/s41366-018-0113-2 29795472

[B28] Stojanov S. Berlec A. (2020). The Influence of Probiotics on the Firmicutes/Bacteroidetes Ratio in the Treatment of Obesity and Inflammatory Bowel Disease. Microorganisms 8, 1715. 10.3390/microorganisms8111715 PMC7692443 33139627

[B29] Tauqeer Z. Gomez G. Stanford F. C. (2018). Obesity in Women: Insights for the Clinician. J. Womens Health 27, 444–457. 10.1089/jwh.2016.6196 PMC6110123 29077514

[B30] Wang H. Hu C. Cheng C. Cui J. Ji Y. Hao X.. (2019). Unraveling the Association of Fecal Microbiota and Oxidative Stress With Stillbirth Rate of Sows. Theriogenology 136, 131–137. 10.1016/j.theriogenology.2019.06.028 31255919

[B31] Wu F. Tian F. J. Lin Y. Xu W. M. (2016). Oxidative Stress: Placenta Function and Dysfunction. Am. J. Reprod. Immunol. 76, 258–271. 10.1111/aji.12454 26589876

[B32] Xu N. Kan P. Yao X. Yang P. Wang J. Xiang L.. (2018). Astragaloside Iv Reversed the Autophagy and Oxidative Stress Induced by the Intestinal Microbiota of Ais in Mice. J. Microbiol. 56, 838–846. 10.1007/s12275-018-8327-5 30353470

[B33] Zhang Y. Sowers J. R. Ren J. (2018). Targeting Autophagy in Obesity: From Pathophysiology to Management. Nat. Rev. Endocrinol. 14, 356–376. 10.1038/s41574-018-0009-1 29686432

[B34] Zhi C. Huang J. Wang J. Cao H. Bai Y. Guo J.. (2019). Connection Between Gut Microbiome and the Development of Obesity. Eur. J. Clin. Microbiol. Infect. Dis. 38, 1987–1998. 10.1007/s10096-019-03623-x 31367997

[B35] Zhou Y. Xu T. Cai A. Wu Y. Wei H. Jiang S.. (2018). Excessive Backfat of Sows at 109 D of Gestation Induces Lipotoxic Placental Environment and Is Associated With Declining Reproductive Performance. J. Anim. Sci. 96, 250–257. 10.1093/jas/skx041 29385477 PMC6145413

