# Supplementary material for: Maternal Obesity Increases Oxidative Stress in Placenta and It Is Associated With Intestinal Microbiota
Source: Front Cell Infect Microbiol. 2021 Aug 23;11:671347. doi: 10.3389/fcimb.2021.671347 (PMC8420882; doi:10.3389/fcimb.2021.671347)
Supplement: Supplementary file 1 [file Table_1.docx]

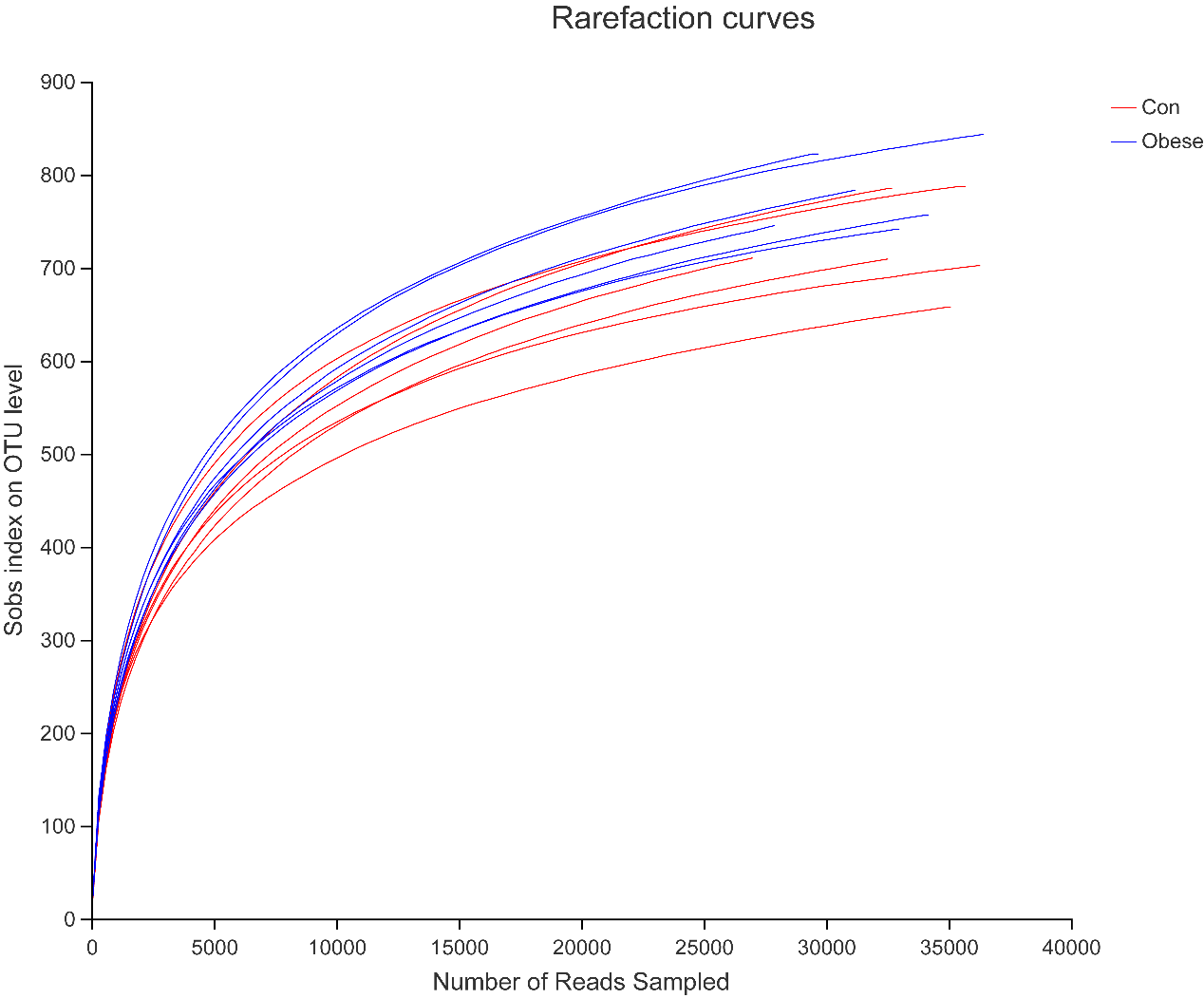


Supplemental Figure 1. Rarefaction curves based on OUT levels.


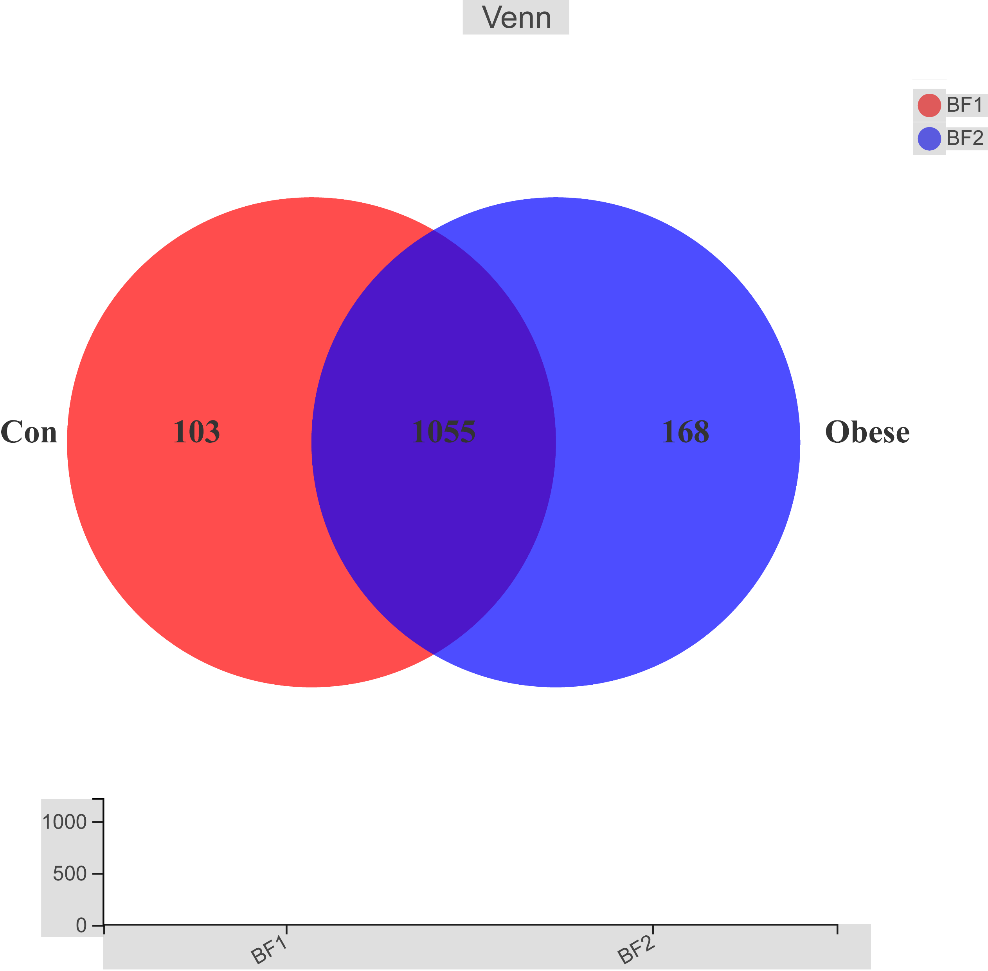


Supplemental Figure 2. The venn diagram for the shared and unique OTUs.

Supplemental table 1. Primers used for real-time PCR

| Genes | Primers | Sequences (5'to 3') |
| --- | --- | --- |
| IL-6 | Forward | AATGTCGAGGCTGTGCAGATT-3 |
|  | Reverse | TGGTGGCTTTGTCTGGATTCT |
| IL-1β | Forward | GCCAGTCTTCATTGTTCAGGTTT |
|  | Reverse | TTGTCACCGTAGTTAGCCATCACT |
| TNF-α | Forward | TGGCCCCTTGAGCATCA |
|  | Reverse | CGGGCTTATCTGAGGTTTGAGA |
| 18S rRNA | Forward | GGTAGTGACGAAAAATAACAATACAGGAC |
|  | Reverse | ATACGCTATTGGAGCTGGAATTACC |

Supplemental table 2. Mean count of raw sequences among the 2 groups

| Items | Con group | Obese group |
| --- | --- | --- |
| No. sequences | 39,304 | 40,224 |
